# The G-quadruplex-forming aptamer AS1411 potently inhibits HIV-1 attachment to the host cell

**DOI:** 10.1016/j.ijantimicag.2016.01.016

**Published:** 2016-04

**Authors:** Rosalba Perrone, Elena Butovskaya, Sara Lago, Alfredo Garzino-Demo, Christophe Pannecouque, Giorgio Palù, Sara N. Richter

**Affiliations:** a Department of Molecular Medicine, University of Padua, Padua, Italy; b Institute of Human Virology, University of Maryland School of Medicine, Baltimore, MD 21201, USA; c KU Leuven, Department of Microbiology and Immunology, Laboratory of Virology and Chemotherapy, Rega Institute for Medical Research, B-3000 Leuven, Belgium

**Keywords:** HIV, Aptamer, Antiretroviral drug, Attachment

## Abstract

AS1411 is a G-rich aptamer that forms a stable G-quadruplex structure and displays antineoplastic properties both in vitro and in vivo. This oligonucleotide has undergone phase 2 clinical trials. The major molecular target of AS1411 is nucleolin (NCL), a multifunctional nucleolar protein also present in the cell membrane where it selectively mediates the binding and uptake of AS1411. Cell-surface NCL has been recognised as a low-affinity co-receptor for human immunodeficiency virus type 1 (HIV-1) anchorage on target cells. Here we assessed the anti-HIV-1 properties and underlying mechanism of action of AS1411. The antiviral activity of AS1411 was determined towards different HIV-1 strains, host cells and at various times post-infection. Acutely, persistently and latently infected cells were tested, including HIV-1-infected peripheral blood mononuclear cells from a healthy donor. Mechanistic studies to exclude modes of action other than virus binding via NCL were performed. AS1411 efficiently inhibited HIV-1 attachment/entry into the host cell. The aptamer displayed antiviral activity in the absence of cytotoxicity at the tested doses, therefore displaying a wide therapeutic window and favourable selectivity indexes. These findings, besides validating cell-surface-expressed NCL as an antiviral target, open the way for the possible use of AS1411 as a new potent and promisingly safe anti-HIV-1 agent.

## Highlights

• The G-quadruplex-forming aptamer AS1411 strongly inhibits HIV-1 infection.
• AS1411 is non-toxic to the host cell at antiviral concentrations.
• AS1411 blocks viral attachment to the host cell.
• AS1411 binds cell-surface-expressed nucleolin, a putative HIV-1 co-receptor.

## 1 Introduction

G-quadruplexes are guanine (G)-rich oligonucleotides capable of forming four-stranded structures. Among G-quadruplex-forming G-rich oligonucleotides, the aptamer AS1411 has stood out for its improved biological activity. AS1411 was described in the late 1990s [1] and was later developed as an anticancer agent. The mechanism of action of AS1411 has been ascribed to its binding to the multifunctional cellular protein nucleolin (NCL). NCL is overexpressed on cancer cell membranes and mediates AS1411 cellular uptake [2]; in turn, selective binding between AS1411 and NCL affects intracellular NCL-dependent pathways, resulting in the biological effects of AS1411 [2], [3], [4], [5].

AS1411 has now completed two phase 2 clinical trials. The results were encouraging with regard to toxicity since the aptamer was very well tolerated with no severe adverse events related to drug administration. However, the anticancer activity was limited to one patient; the basis for such activity is currently under investigation [6].

Interestingly, NCL is involved in the very initial step of human immunodeficiency virus type 1 (HIV-1) virion–cell recognition, referred to as attachment. HIV-1 attachment can occur in the absence of the main cellular receptors, i.e. CD4 and CXCR4/CCR5, through co-ordinated interactions with heparan sulfate proteoglycans and cell-surface-expressed NCL [7]. NCL was found in cellular fractions containing the HIV genome, viral matrix and reverse transcriptase as well as in cross-linked complexes with CD4 and CXCR4/CCR5 at the cell membrane, supporting its potential role in viral entry [8], [9]. Cell-surface NCL is markedly downregulated immediately after HIV entry into permissive cells as a consequence of its translocation into the cytoplasm [8]. Moreover, NCL is involved in specific HIV-associated cell cycle perturbations since lymphocytes isolated from HIV-infected patients displayed NCL hyperlocalisation on the cell surface [10]; in patients treated with antiretroviral therapy this effect was also observed in HIV-infected subjects with poor immunological recovery [11]. Thereafter, cell surface NCL has been proposed as an anti-HIV-1 target: a pentameric pseudopeptide [8] and natural NCL ligands located in the cell membrane [12], [13], [14] indeed inhibited HIV replication by blocking virus attachment to the cell surface. In the recent years, cell membrane molecules such as chemokine co-receptors CXCR4 and CCR5 have emerged as attractive therapeutic targets for anti-HIV-1 therapy since the use of specific antagonists efficiently blocked the HIV-1 entry process. For example, the CCR5 antagonist maraviroc has been approved by the US Food and Drug Administration (FDA) to treat HIV-1-infected persons and has proved extremely effective [15].

Based on the above information, the aim of this study was to test whether the aptamer AS1411 was able to interfere with HIV-1 cellular entry. An indication of the antiviral activity of AS1411 has recently been provided [16]. Here we aimed at testing AS1411 against different viral strains, cell lines and primary cells and investigating the most relevant mechanism of action.

## 2 Materials and methods

### 2.1 Oligonucleotides and compounds

Oligonucleotides (Supplementary Table S1), dextran sulfate 8000 (DS 8000) and AMD3100 were purchased from Sigma-Aldrich (Milan, Italy), nevirapine was from Boehringer Ingelheim (Ridgefield, CN) and enfuvirtide (Fuzeon^®^) was from Roche (Vilvoorde, Belgium). Zidovudine (AZT) was synthesised by one of the authors (CP).

Supplementary Table S1 related to this article can be found, in the online version, at http://dx.doi.org/10.1016/j.ijantimicag.2016.01.016.

Table S1 Oligonucleotides used in antiviral assays and surface plasmon resonance (SPR) analysis.

### 2.2 Virus stocks

HIV-1_NL4-3_ and HIV-1_BaL_ stocks were prepared by transfection of HEK 293T with proviral genomes (NIH AIDS Research and Reference Reagent Program). The HIV-1_IIIB_ stock was originally provided by Prof. R.C. Gallo and Dr M. Popovic.

### 2.3 Antiviral assay

The TZM-bl reporter cell line (NIH AIDS Research and Reference Reagent Programme) was seeded in 96-well plates (Falcon^®^; Corning Life Sciences, Durham, NC), was infected with HIV-1_NL4-3_ or HIV-1_BaL_ at different multiplicities of infection (MOIs) (0.05, 0.1 and 0.5) and was treated with serial dilutions of test oligonucleotides. After 24–72 h, HIV-1 production was assessed following the long terminal repeat (LTR) luciferase signal using the britelite™ plus Reporter Gene Assay System (Perkin Elmer, Waltham, MA) according to the manufacturer's protocol.

MT-4 cells (NIH AIDS Research and Reference Reagent Program) were seeded in 96-well plates in the presence of HIV-1_NL4-3_ at different MOIs (0.003, 0.05, 0.1 and 0.5) and serial dilutions of AS1411 or CRO26. After 24–120 h, HIV-1 production was determined by measuring p24 antigen concentration in the supernatant using an HIV-1 p24 ELISA assay (XpressBio, Thurmont, MD). Sample absorbance at 450 nm was measured using a Tecan Sunrise™ Microplate Reader (Tecan, Cernusco sul Naviglio, Italy). The cytotoxicity of test oligonucleotides in TZM-bl and MT-4 cells was tested using the MTT [(3-(4,5-dimethylthiazol-2-yl)-2,5-diphenyltetrazolium bromide) tetrazolium] assay.

Peripheral blood mononuclear cells (PBMCs) were activated for 48 h with phytohaemagglutinin (2.5 μg/mL) and interleukin-2 (IL-2) (10 ng/mL) and were washed and cultured in complete RPMI-1640 and IL-2 (10 ng/mL). Activated PBMCs were infected for 2 h with HIV_IIIB_ (using 5.5 ng equivalent of p24 per 10^5^ cells) and were untreated or treated with AS1411, CRO26, AMD3100 or enfuvirtide. AS1411 and CRO26 were replenished every 28 h. Aliquots of supernatants were obtained at Days 4, 6, 8 and 10 post-infection (p.i.) and p24 was measured. At Day 10 p.i., cells were counted using trypan blue exclusion to assess viability, and MTS [3-(4,5-dimethylthiazol-2-yl)-5-(3-carboxymethoxyphenyl)-2-(4-sulfophenyl)-2H-tetrazolium] assays were performed to evaluate cell metabolism.

### 2.4 Antiviral assay in chronically HIV-1-infected cells

HuT78/IIIB cells are HuT78 cells persistently infected with the HIV-1_IIIB_ strain. The antiviral activities of test aptamers against persistent HIV-1 infection were based on inhibition of p24 antigen production in Hut78/IIIB cells pre-treated with AZT (10 ng/mL). Pre-treated HuT78/IIIB cells were seeded in a 48-well plate (Costar^®^; Corning Inc., Corning, NY) in the presence of serial dilutions of AS1411 or CRO26. After 43 h, supernatants were collected and HIV-1 production was determined by measuring p24 antigen. The cytotoxicity of test aptamers on HuT78/IIIB cells was tested in parallel by MTT assay.

### 2.5 Antiviral assay in latently infected cells

OM10.1 (NIH AIDS Research and Reference Reagent Program) is a promyelocytic HIV-1 latently infected HL-60 cell line. The antiviral activity of test aptamers was based on inhibition of p24 antigen production in OM10.1 cells pre-treated with AZT (10 ng/mL) and stimulated with phorbol-12-myristate-13-acetate (PMA) (0.02 μM). Cells were seeded in a 48-well plate in the presence of serial dilutions of AS1411 or CRO26. After 48 h, supernatants were collected and HIV-1 production was determined by measuring p24 antigen as previously described. The cytotoxicity of test aptamers on OM10.1 cells was assessed in parallel using the MTT assay.

### 2.6 Viral binding assay

MT-4 cells were incubated with serial dilutions of test aptamers, DS 8000 or AMD3100 and then HIV-1_IIIB_ stock dilutions (corresponding to 100 ng of p24) were added. After 2 h at 37 °C, cells were washed to remove unbound viral particles and were subsequently lysed with 1× phosphate-buffered saline (PBS) containing 0.5% Tergitol^®^ NP-40 (Sigma, St Louis, MO). The amount of p24 antigen in the cell lysate was measured as described above.

### 2.7 Time-of-addition assay

MT-4 cells were infected with HIV-1_IIIB_ at a MOI of 0.5. After 1 h, cells were washed, seeded and incubated at 37 °C. AS1411 and the reference compounds DS 8000, AMD3100, enfuvirtide, AZT and nevirapine were added at different time points p.i. AS1411 was added at 25 μM and the reference compounds were added at a concentration corresponding to 100-fold their 50% inhibitory concentration (IC_50_). HIV-1 production was determined 31 h p.i. by measuring p24 antigen in the supernatant.

### 2.8 Surface plasmon resonance (SPR)-directed affinity binding analysis

SPR experiments were performed on a Biacore™ T100 platform (GE Healthcare Europe, Milan, Italy). The human recombinant protein NCL (OriGene Technologies Inc., Rockville, MD) and the HIV-1_IIIB_ recombinant gp120 (ImmunoDiagnostics Inc., Woburn, MA) were immobilised on a Series S Sensor Chip CM5 (GE Healthcare Europe) by amine coupling in HEPES-NaCl running buffer. NCL and gp120 were diluted in sodium acetate buffer at 15 ng/μL and 20 ng/μL, respectively, and were injected to reach 1500 resonance units (RU). Binding analysis was performed at 20 μL/min with contact and dissociation times of 120 s in HEPES-KCl buffer. Test oligonucleotides (Supplementary Table S1) were diluted to 1 μM in HEPES-KCl buffer, were denatured at 95 °C for 5 min and were cooled at room temperature. Sensorgrams were obtained in the concentration range of 15.6–1000 nM. The chip surface was regenerated with 1 M KCl solution. All sensorgrams were corrected by subtraction of blank flow cell response and buffer injection response.

## 3 Results

### 3.1 The AS1411 aptamer has potent anti-HIV-1 activity against different strains in cell lines and primary cells

Based on the known role of NCL in HIV attachment, we investigated whether AS1411 was able to inhibit HIV-1 replication by blocking its attachment/entry into the host cell through interaction with cell-surface NCL. The antiviral activity of AS1411 was initially assessed in TMZ-bl and MT-4 cell lines, which were infected with X4/R5-tropic HIV-1 strains at different MOIs (0.003, 0.05, 0.1 and 0.5) and were incubated in the presence of serial dilutions of AS1411 or the negative control CRO26. Virus amount in the supernatant was assessed at different time points p.i. (24, 48, 72 and 120 h p.i.) through quantification of the luciferase signal (TZM-bl) or p24 viral protein (MT-4). The cytotoxicity of AS1411 and CRO26 was assessed in parallel using the MTT assay. AS1411 markedly reduced virus production in all tested conditions (Table 1), whilst CRO26 did not show any antiviral effect (Supplementary Table S2). A slight decrease in the antiretroviral activity of AS1411 was observed at increasing times p.i. (i.e. 72 h and 120 h p.i.). Because the aptamer was administrated only once at the time of infection, we hypothesised that reduction of compound effectiveness [17] was the main cause of the decreased antiviral activity at long test times. To test this hypothesis, AS1411 was administered at 24-h intervals for four times in infected MT-4 cells and virus production was measured at 120 h p.i. An increase in AS1411 efficacy was indeed observed compared with the single administration of the aptamer (Table 1). Notably, AS1411 had no cytotoxicity up to the highest tested concentration, yielding high selectivity indexes of >500 under most tested conditions.

Supplementary Table S2 related to this article can be found, in the online version, at http://dx.doi.org/10.1016/j.ijantimicag.2016.01.016.

Table S2 Anti-HIV-1 activity and cytotoxicity of control oligonucleotides CRO26, LTR-III and SCRA.

**Fig. 1:**
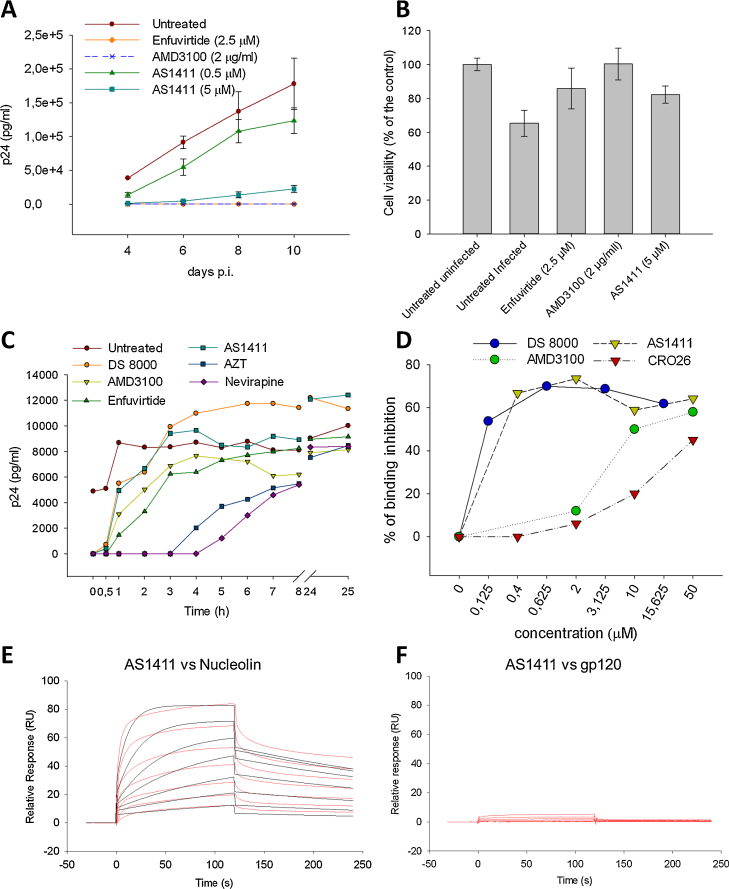
Inhibition of a human immunodeficiency virus type 1 (HIV-1) early step by AS1411. (A) Antiviral activity of AS1411 in HIV-1_IIIB_-infected peripheral blood mononuclear cells (PBMCs). Freshly isolated PBMCs were infected with HIV-1_IIIB_ and treated with test aptamer or reference compounds. Virus-associated p24 antigen was measured at Days 4, 6, 8 and 10 post-infection (p.i.). (B) Cell viability evaluated via MTS assay at 10 days p.i. in HIV-1_IIIB_-infected PBMCs and treated with AS1411 or reference compounds. (C) Time-of-addition assay. MT-4 cells were infected with HIV-1_IIIB_ and test aptamer or reference compounds were added at different time points after infection. Virus-associated p24 antigen in the supernatant was measured 31 h p.i. The antiviral activity of AS1411 was compared with that of the negative control and with reference drugs. The data are representative of two independent experiments. (D) Viral binding assay. Effect of AS1411 on HIV-1_IIIB_ binding to MT-4 cells. Cells were incubated with HIV-1_IIIB_ viral particles in the presence or absence of different concentrations of AS1411 and were subsequently cleared from excess compound by washing steps. DS 8000 and AMD3100 were tested in parallel as reference drugs. After lysis of the cells and eventually attached virus, the amount of virion-associated p24 was quantified and used to calculate the percent of binding inhibition. (E, F) Surface plasmon resonance (SPR) plots of AS1411 (concentrations 15.6, 31.2, 62.5, 125, 250, 500 and 1000 nM) interactions with nucleolin (E) or HIV-1_IIIB_ gp120 (F). Experimental curves in red and fitting curves in black. (For interpretation of the references to colour in this figure legend, the reader is referred to the web version of this article.)

AS1411 was next evaluated in freshly isolated PBMCs, a natural target during in vivo infection. When PBMCs were infected with HIV-1_IIIB_, AS1411 inhibited viral production by ca. 64% and 97% at 500 nM and 5 μM, respectively, at 4 days p.i. (Fig. 1A). Consistent with previous results, a decrease in activity was observed at increasing times p.i. However, at 10 days p.i. and 5 μM, AS1411 retained significant antiviral activity with 80% inhibition of viral production (Fig. 1A) and prevented viral-induced cell death as shown by the MTS assay (Fig. 1B). Similar results were obtained with the reference HIV-1 entry inhibitors enfuvirtide and AMD3100 (Fig. 1A, B). These data indicate that the antiviral activity of AS1411 is relevant to primary infected cells.

To assess the mechanism of action of AS1411, two infected cell models sensitive to drugs acting during post-integration steps were investigated. OM-10.1 cells are latently HIV-1-infected promyelocytes containing a single integrated provirus; they sustain latent HIV-1 infection under normal culture conditions. HIV-1 production is induced by phorbol esters (e.g. PMA). HuT78/IIIB cells are HIV-1_IIIB_-persistently infected HuT78 cells stably producing infectious viral particles. AZT pre-treatment of HuT78/IIIB cells allows the evaluation of post-integration effects. In both setups, treatment with AS1411 did not produce antiviral effects as measured by p24 antigen production (Table 1), indicating that no post-integration events were affected by AS1411.

The viral step targeted by AS1411 was investigated by a time-of-addition assay. After infection, AS1411 and control compounds were added each hour over 8 h and at 24–25 h p.i. and virus replication was monitored by p24 expression at 31 h p.i. AS1411 had to be added at the time of infection (0 h p.i.) to observe inhibitory activity on virus production. This behaviour was shared with the attachment inhibitor DS 8000 and was similar to that of the CXCR4 co-receptor antagonist AMD3100 (Fig. 1C). In contrast, the fusion inhibitor enfuvirtide maintained activity when added up to 30 min after infection. These data suggest that AS1411 inhibits adsorption of the virus to the host cell. This mechanism of action was confirmed by a viral binding assay. AS1411 was able to block viral binding by 65% with respect to the untreated control with an IC_50_ value lower than 400 nM, whilst the control oligonucleotide CRO26 inhibited viral binding up to 45% only at the highest tested concentration of 50 μM, probably owing to a reported general mechanism of viral entry inhibition due to an unspecific mechanism of electrostatic interaction with the viral/cell surface [18] (Fig. 1D). Both DS 8000 and AMD3100 blocked virus binding up to 65% and 58%, respectively, of the untreated control (Fig. 1D). The aptamer behaviour was again superimposable to that of DS 8000, indicating that virus attachment is the main targeted step.

Some anti-HIV-1 G-quadruplex (G4)-forming aptamers have been reported to bind the HIV-1 gp120 envelope glycoprotein, blocking virus attachment [18]. We thus investigated whether gp120 was targeted by AS1411 by measuring binding affinity by SPR. We also tested a HIV-1 G4-forming oligonucleotide (LTR-III) [19], a G-rich non-G4-forming scrambled oligonucleotide (SCRA) and the C-rich sequence CRO26. Although some G4 structures have been reported to bind gp120 [20], the binding to gp120 of both AS1411 and LTR-III was too low to obtain meaningful binding affinity constants (Fig. 1F; Supplementary Fig. S1B). The other sequences did not bind as well (Supplementary Fig. S1D, F). Binding to NCL was next assessed for comparison. AS1411 showed a dose-dependent interaction with NCL (*K*_D_ = 34.2 nM) with fast association rates (Fig. 1E); in this setting, LTR-III showed lower binding affinity to NCL (*K*_D_ = 76.3 nM) and slower association rates compared with AS1411 (Supplementary Fig. S1A). In contrast, SCRA and CRO26 exhibited very low binding to NCL and no *K*_D_ could be calculated in these conditions (Supplementary Fig. S1C, E). The antiviral activity of LTR-III was also assessed and was ca. 40 times lower than that of AS1411, whereas SCRA was inactive (Supplementary Table S2). These data support that binding to NCL is the main mechanism of action of AS1411: the peculiar binding mode of AS1411, due to its dimeric nature, high stability and natural propensity to fold into G4, may account for the improved antiviral activity compared with other G4-forming oligonucleotides.

Supplementary Fig. S1 related to this article can be found, in the online version, at http://dx.doi.org/10.1016/j.ijantimicag.2016.01.016.

Fig. S1 Surface plasmon resonance (SPR) binding analysis of nucleolin (NCL) and gp120 with control oligonucleotides LTR-III, CRO26 and SCRA. (A, B) SPR sensorgrams of the binding of the G-quadruplex (G4)-forming oligonucleotide LTR-III (concentrations of 15.6, 31.2, 62.5, 125, 250, 500 and 1000 nM) to nucleolin (A) or HIV-1_IIIB_ gp120 (B). (C, D) SPR sensorgrams of the binding of CRO26 (concentrations of 15.6, 31.2, 62.5, 125, 250, 500 and 1000 nM) to nucleolin (C) or HIV-1_IIIB_ gp120 (D). (E, F) SPR sensorgrams of the binding of the G-rich SCRA oligonucleotide (concentrations of 15.6, 31.2, 62.5, 125, 250, 500 and 1000 nM) to nucleolin (E) or HIV-1_IIIB_ gp120 (F). Experimental curves in red and fitting curves in black.

## 4 Discussion

Aptamers, short oligonucleotides that adopt specific three-dimensional structures in vivo, are able to bind with high affinity to target biomolecules and elicit a biological response. Their binding affinities to the target are generally similar to those of antibodies. However, unlike antibodies, they are extremely stable over temperature, with solvent exposure and in harsh environments; they lack immunogenicity; and they display low interbatch variability. Owing to their small size they penetrate tissues fast and are rapidly cleared from the blood. With these attractive features, aptamers have been intensely investigated as therapeutics. One aptamer (Macugen) has reached the clinic for the treatment of age-related macular degeneration by targeting vascular endothelial growth factor. Five other aptamers, among which is AS1411, have been tested in phase 2 clinical trials [21]. In the HIV field, most aptamers have been tested against viral proteins (i.e. gp120, reverse transcriptase, integrase) but none proceeded to advanced clinical trials [20], [22], [23], [24], [25], likely due to target mutations or pharmacokinetic limits, shared by all aptamers. Interestingly, the G4 folding of AS1411 makes it more resistant to endonucleases [26]. We and others have previously shown that the HIV-1 LTR promoter forms a dynamic G4 structure [19], [27] that can be targeted by G4 ligands to block viral transcription [28], [29]. Interaction of the LTR G4s with NCL also strongly inhibits transcription [30]. We initially tested AS1411 to investigate whether its reported sequestration of cellular NCL could affect LTR activity. In contrast, we found that AS1411 significantly inhibited virus attachment. The equally high activity of the aptamer against X4- and R5-tropic HIV-1 strains indicates that the effect is independent of the nature of the ‘classic’ co-receptors. The fact that AS1411 strongly binds NCL (SPR data), its reported binding and intracellular uptake of membrane NCL [1] and the evidence of NCL usage by HIV as a co-receptor [7] suggest a mechanism mediated by AS1411 interference at NCL that blocks virus attachment.

For the antiviral activity of AS1411, lower doses and fewer administrations are required than for the aptamer's antineoplastic activity [1], [2], [31], [32], therefore showing a broad potential therapeutic window for antiviral purposes. Moreover, if the anticancer activity of AS1411 is confirmed, the recently reported acquisition of cancer features in HIV-1-infected cells during antiretroviral therapy treatment [33] might also be targeted, with the fascinating possibility to exploit the anticancer effects of AS1411 during HIV-1 treatment, for example in seropositive patients affected by AIDS-related cancers.

## 5 Conclusions

We have shown that AS1411, a G4-forming oligonucleotide, has the potential to block HIV-1 infection by binding to cell surface NCL and interfering with HIV-1 cell attachment. Since AS1411 has already shown mild toxicity in clinical trials, testing in humans as an anti-HIV-1 agent with a new mechanism of action can be envisaged in the near future.

## Figures and Tables

**Table 1:** Anti-HIV-1 activity and cytotoxicity of the G-quadruplex-forming aptamer AS1411.

Hours p.i.	Cell line	Virus	Strain	Tropism	Type of infection	IC_50_ (nM)^a^	CC_50_ (nM)^b^	SI^c^	Administration times
24	TZM-bl	HIV-1	NL4-3	X4 tropic	Ex novo	13.7 ± 1.7	>25 000	>1825	1
MT-4	HIV-1	NL4-3	X4 tropic	Ex novo	16.2 ± 4.3	>25 000	>1543	1
TZM-bl	HIV-1	BaL	R5 tropic	Ex novo	19.6 ± 3.8	>25 000	>1276	1

48	TZM-bl	HIV-1	NL4-3	X4 tropic	Ex novo	15.3 ± 1.9	>25 000	>1634	1
MT-4	HIV-1	NL4-3	X4 tropic	Ex novo	23.7 ± 3.7	>25 000	>1055	1
TZM-bl	HIV-1	BaL	R5 tropic	Ex novo	36.5 ± 3.0	>25 000	>685	1

72	TZM-bl	HIV-1	NL4-3	X4 tropic	Ex novo	22.3 ± 3.0	>25 000	>1121	1
MT-4	HIV-1	NL4-3	X4 tropic	Ex novo	105.3 ± 16.9	>25 000	>237	1
TZM-bl	HIV-1	BaL	R5 tropic	Ex novo	45.4 ± 5.2	>25 000	>551	1

120	MT-4	HIV-1	NL4-3	X4 tropic	Ex novo	1490.9 ± 50.1	>5000	>3	1
MT-4	HIV-1	NL4-3	X4 tropic	Ex novo	405.7 ± 19.0	>5000	>12	4
–	HuT78/IIIB	HIV-1	IIIB	X4 tropic	Persistent	>25 000	>25 000	–	1
–	OM10.1	HIV-1	LAI	X4 tropic	Latent	>25 000	>25 000	–	1

a 50% inhibitory concentration, defined as the concentration of the oligonucleotide required to inhibit HIV-1 production by 50%, as evaluated by long terminal repeat (LTR) luciferase signal (TZM-bl) or p24 antigen production (MT-4).

b 50% cytotoxic concentration, defined as the concentration of the oligonucleotide required to reduce cell proliferation by 50%.

c The selectivity index (SI) is the relative effectiveness of the tested aptamer in inhibiting viral replication compared with inducing cell death (CC_50_/IC_50_).
