# Supplementary material for: The G-quadruplex-forming aptamer AS1411 potently inhibits HIV-1 attachment to the host cell
Source: Int J Antimicrob Agents. 2016 Apr;47(4):311–6. doi: 10.1016/j.ijantimicag.2016.01.016 (PMC4840014; doi:10.1016/j.ijantimicag.2016.01.016)
Supplement: Table S1 — Oligonucleotides used in antiviral assays and surface plasmon resonance (SPR) analysis. [file mmc1.docx]

**Supplementary Table S1**

Oligonucleotides used in antiviral assays and surface plasmon resonance (SPR) analysis

| **Name** | **Sequence (5’–3’)** |
| --- | --- |
| AS1411 | GGTGGTGGTGGTTGTGGTGGTGGTGG |
| CRO26 | CCTCCTCCTCCTTCTCCTCCTCCTCC |
| LTR-III | GGGAGGCGTGGCCTGGGCGGGACTGGGG |
| SCRA | TTTTTGGAGCGTGTGTGCGCGAGAGCGTGCGCGTGGCGAGCGTGGAGTGGTTTTT |
