# Supplementary material for: The G-quadruplex-forming aptamer AS1411 potently inhibits HIV-1 attachment to the host cell
Source: Int J Antimicrob Agents. 2016 Apr;47(4):311–6. doi: 10.1016/j.ijantimicag.2016.01.016 (PMC4840014; doi:10.1016/j.ijantimicag.2016.01.016)
Supplement: Table S2 — Anti-HIV-1 activity and cytotoxicity of control oligonucleotides CRO26, LTR-III and SCRA. [file mmc2.docx]

**Supplementary Table S2**

Anti-HIV-1 activity and cytotoxicity of control oligonucleotides CRO26, LTR-III and SCRA

| **Oligonucleotide** | **Hours p.i.** | **Cell line** | **Virus** | **Strain** | **Tropism** | **Type of infection** | **IC_50_ (μM) ^a^** | **CC_50_ (μM) ^b^** | **SI ^c^** | **Administration times** |
| --- | --- | --- | --- | --- | --- | --- | --- | --- | --- | --- |
| CRO26 | 24 | TZM-bl | HIV-1 | NL4-3 | X4 tropic | Ex novo | >25 | >25 | – | 1 |
|  |  | MT-4 | HIV-1 | NL4-3 | X4 tropic | Ex novo | >25 | >25 | – | 1 |
|  |  | TZM-bl | HIV-1 | BaL | R5 tropic | Ex novo | >25 | >25 | – | 1 |
|  | 48 | TZM-bl | HIV-1 | NL4-3 | X4 tropic | Ex novo | >25 | >25 | – | 1 |
|  |  | MT-4 | HIV-1 | NL4-3 | X4 tropic | Ex novo | >25 | >25 | – | 1 |
|  |  | TZM-bl | HIV-1 | BaL | R5 tropic | Ex novo | >25 | >25 | – | 1 |
|  | 72 | TZM-bl | HIV-1 | NL4-3 | X4 tropic | Ex novo | >25 | >25 | – | 1 |
|  |  | MT-4 | HIV-1 | NL4-3 | X4 tropic | Ex novo | >25 | >25 | – | 1 |
|  |  | TZM-bl | HIV-1 | BaL | R5 tropic | Ex novo | >25 | >25 | – | 1 |
|  | 120 | MT-4 | HIV-1 | NL4-3 | X4 tropic | Ex novo | >5 | >5 | – | 1 |
|  |  | MT-4 | HIV-1 | NL4-3 | X4 tropic | Ex novo | >5 | >5 | – | 4 |
|  | – | HuT78/IIIB | HIV-1 | IIIB | X4 tropic | Persistent | >25 | >25 | – | 1 |
|  | – | OM10.1 | HIV-1 | LAI | X4 tropic | Latent | >25 | >25 | – | 1 |
| LTR-III | 24 | TZM-bl | HIV-1 | NL4-3 | X4 tropic | Ex novo | 0.57 ± 0.05 | >5 | – | 1 |
| SCRA | 24 | TZM-bl | HIV-1 | NL4-3 | X4 tropic | Ex novo | >5 | >5 | – | 1 |

p.i., post-infection.

^a^ 50% inhibitory concentration, defined as the concentration of the oligonucleotide required to inhibit HIV-1 production by 50%.

^b^ 50% cytotoxic concentration, defined as the concentration of the oligonucleotide required to reduce cell proliferation by 50%.

^c^ The selectivity index (SI) for HIV-1, calculated as the ratio of CC_50_/IC_50_.
