# Supplementary material for: The G-quadruplex-forming aptamer AS1411 potently inhibits HIV-1 attachment to the host cell
Source: Int J Antimicrob Agents. 2016 Apr;47(4):311–6. doi: 10.1016/j.ijantimicag.2016.01.016 (PMC4840014; doi:10.1016/j.ijantimicag.2016.01.016)
Supplement: Fig. S1 — Surface plasmon resonance (SPR) binding analysis of nucleolin (NCL) and gp120 with control oligonucleotides LTR-III, CRO26 and SCRA. (A, B) SPR sensorgrams of the binding of the G-quadruplex (G4)-forming oligonucleotide LTR-III (concentrations of 15.6, 31.2, 62.5, 125, 250, 500 and 1000 nM) to nucleolin (A) or HIV-1IIIB gp120 (B). (C, D) SPR sensorgrams of the binding of CRO26 (concentrations of 15.6, 31.2, 62.5, 125, 250, 500 and 1000 nM) to nucleolin (C) or HIV-1IIIB gp120 (D). (E, F) SPR sensorgrams of the binding of the G-rich SCRA oligonucleotide (concentrations of 15.6, 31.2, 62.5, 125, 250, 500 and 1000 nM) to nucleolin (E) or HIV-1IIIB gp120 (F). Experimental curves in red and fitting curves in black. [file mmc3.docx]

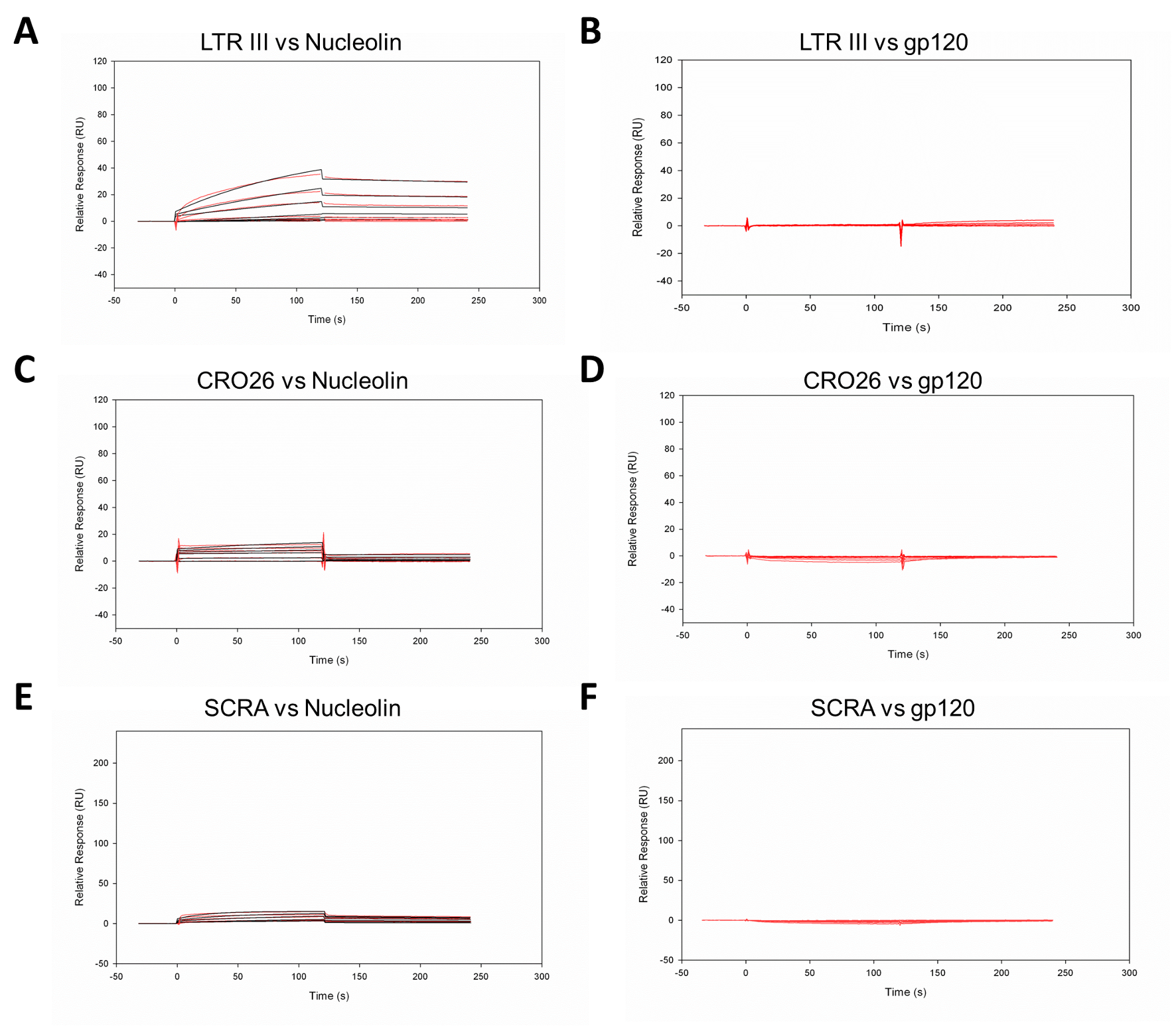


**Supplementary Fig. S1.** Surface plasmon resonance (SPR) binding analysis of nucleolin (NCL) and gp120 with control oligonucleotides LTR-III, CRO26 and SCRA. (A,B) SPR sensorgrams of the binding of the G-quadruplex (G4)-forming oligonucleotide LTR-III (concentrations of 15.6, 31.2, 62.5, 125, 250, 500 and 1000 nM) to nucleolin (A) or HIV-1_IIIB_ gp120 (B). (C,D) SPR sensorgrams of the binding of CRO26 (concentrations of 15.6, 31.2, 62.5, 125, 250, 500 and 1000 nM) to nucleolin (C) or HIV-1_IIIB_ gp120 (D). (E,F) SPR sensorgrams of the binding of the G-rich SCRA oligonucleotide (concentrations of 15.6, 31.2, 62.5, 125, 250, 500 and 1000 nM) to nucleolin (E) or HIV-1_IIIB_ gp120 (F). Experimental curves in red and fitting curves in black.
